# Theoretical Investigation of the Functionalized MOene V_2_O: A Density Functional Approach

**DOI:** 10.1021/acsomega.6c00785

**Published:** 2026-04-21

**Authors:** Santiago Triana Bejarano, Do Minh Hoat, Jonathan Guerrero Sanchez, Carlos Antonio Corona Garcia

**Affiliations:** † Smalley-Curl Institute, 539850 Rice University, Houston, Texas 77005, United States; ‡ Department of Material Science and Nanoengineering, Rice University, Houston, Texas 77005, United States; § Institute of Theoretical and Applied Research, 374802 Duy Tan University, Hanoi 100000, Vietnam; ∥ School of Engineering and Technology, Duy Tan University, Da Nang 550000, Vietnam; ⊥ Centro de Nanociencias y Nanotecnología, Universidad Nacional Autónoma de México, Baja California 22860, México

## Abstract

The structural, thermodynamic, dynamical, and electronic properties of pristine and functionalized V_2_O-based MOenes were investigated using density functional theory (DFT). Both 1T and 2H polymorphs were examined and functionalized with T = {Br, Cl, F, N, OH}. Our results reveal that the pristine 1T and 2H V_2_O MOene phases are dynamically and thermodynamically stable. Among the high-symmetry adsorption sites, the HV site, where the functional group is positioned above the farthest vanadium atom, was identified as the most favorable for the 1T phase, while the H site, corresponding to a hollow position at the center of the hexagonal lattice, was found to be the most stable for the 2H phase. Phonon dispersion analyses confirmed that F- and OH- functionalized systems remain dynamically stable in both polymorphs, whereas Br-functionalization is stable only in the 2H phase. Based on cohesive and formation energies, the 2H configurations are thermodynamically more stable than the corresponding 1T phases. Interestingly, Br functionalization induces structural distortions and a metal-to-semiconductor transition, with an indirect band gap of 0.64 eV. Finally, lithium adsorption calculations show that the 2H phase presents favorable adsorption energies (*E*_ads_ ≈ −1.8 to −1.5 eV) with minimal lattice deformation, suggesting excellent reversibility for ion storage. The results establish V_2_O-based MOenes as a new member of the emerging family of stable 2D oxides with tunable electronic properties and potential applications in energy storage and nanoelectronics.

## Graphical abstract

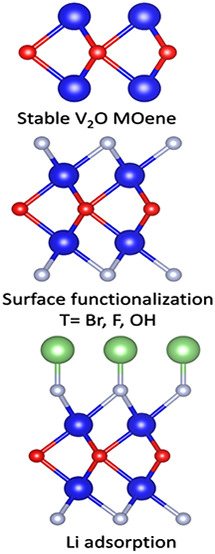

## Introduction

Since the discovery of graphene, two-dimensional (2D) materials have attracted significant scientific interest due to their exceptional physical and chemical properties when compared with their bulk systems.^1,2^ When bulk materials are reduced to the monolayer limit via an exfoliation process,^3,4^ or synthesized 2D materials by CVD,^5,6^ or epitaxially grown atomically thin layers,^7–9^ quantum confinement and reduced dimensionality often lead to remarkable changes in their electronic and structural properties. For example, silicon and germanium in their bulk forms are indirect semiconductors, whereas in their 2D counterparts, silicene and germanene, exhibit distinct electronic dispersions and enhanced tunability.^10–13^ Similarly, single- and few-layer materials such as black phosphorus and transition-metal dichalcogenides exhibit thickness-dependent band structures and carrier-transport properties (mobilities).^14–17^ These reduced-dimensional systems frequently exhibit modified band gaps, enhanced surface-to-volume ratios favorable for catalysis,^18^ tunable electronic structures,^19,20^ improved electronic transport properties,^21^ and increased mechanical flexibility.^22,23^ This fundamental difference between bulk and layered materials has motivated the exploration of a wide variety of 2D materials, including silicene,^10,11^ germanene,^12,13^ the family of phosphorene,^24–26^ the transition-metal dichalcogenides,^27^ and, more recently, the family of MXenes.^28,29^

MXenes are a class of 2D transition-metal carbides or nitrides that are derived from layered MX phases through selective chemical etching.^30^ Their general formula is M_*n*+1_ X_*n*_ T_*y*_, where M is a transition metal, and X is a carbon or nitrogen atom, and T denotes surface functional groups such as Br, Cl, F, N, O, and OH.^28^ In 2011, the first MXene, Ti_3_C_2_, was successfully synthesized, marking the beginning of an active research field. Since then, over one hundred MXenes have been reported either experimentally or theoretically.^30,31^ These materials have shown outstanding versatility, making them suitable for a wide range of applications, including energy storage,^32,33^ catalysis,^32,34,35^ sensors^36,37^ and biomedicine, thanks to their high electrical conductivity, tunable surface chemistry, and structural diversity.^38–40^

Inspired by all the theoretical and experimental advances in MXenes, particularly by the work of Michałowski et al.,^41^ where they used secondary-ion mass spectrometry (SIMS) to report the growth of oxycarbides MXenes (in which oxygen atoms partially replace carbon atoms in the sublattice), and by the work of Yandong Ma et al.,^42^ where they theoretically reported the Tl_2_O single-layer, Luo Yan and collaborators^43^ proposed a new type of emerging materials, the MOenes. These 2D materials are similar to MXenes, but the X element is replaced by oxygen (O). This substitution not only modifies the electronic properties, but it is expected to enhance chemical reactivity in the hydrogen evolution reaction (HER),^31^ introduce novel functionalities, like Weyl Fermions,^43^ and improve electronic mobility.^42^

The field of MOenes is new, and much research remains to be done. Most studies focus only on the Ti_2_O systems,^43,44^ while only one theoretical work has investigated the V_2_O_3_ system, a functionalized form of the V_2_O MOene, without reporting the dynamic stability of the pristine phases.^31^ Vanadium-based MOenes are particularly promising due to their potential applications in electrocatalysis, especially for the hydrogen evolution reaction (HER). Using first-principles calculations, Xie et al.^31^ demonstrated that V_2_O_3_ exhibits favorable HER catalytic activity. However, the structural stability and electronic properties of the pristine and functionalized V_2_O MOene remain unexplored.

In this work, we perform a systematic first-principles study of the V_2_O MOene, considering both 2H and 1T polymorphs. We evaluate their thermodynamic and dynamic stability by computing total energies and phonon dispersion spectra. Their electronic properties are characterized by band structure and density-of-states (DOS) analyses. Furthermore, we explore the influence of various functional groups (T = Br, Cl, F, N, OH) on the stability and electronic properties of V_2_O_3_T_2_ based MOenes. The main objective is to identify new stable V-based MOenes as promising candidates for applications as anode materials for ion batteries and as electrocatalysts for energy conversion reactions.

## Methodology

Total energy calculations based on density functional theory (DFT), as implemented in the Vienna Ab Initio Simulation Package (VASP),^45–48^ were performed to investigate the structural, electronic, and dynamical properties of the 2D transition-metal oxide V_2_O MOene. The simulations were carried out using the unit cells of the 1T and 2H phases, each containing three atoms (two V and one O) per layer for the pristine systems, and five atoms (two V, one O, and two atoms of the functional groups) for the functionalized systems. Spin-polarized calculations were initially considered since vanadium is a transition metal with partially filled 3d orbitals. However, the computed magnetic moments converged to zero for V atoms and all other species in both pristine and functionalized systems, indicating a nonmagnetic ground state. Therefore, spin polarization was not included in the final calculations, as it does not affect the electronic or structural properties of either pristine or functionalized MOenes. Additionally, the Hubbard U correction was not considered, since both pristine and functionalized polymorphs exhibit negligible magnetic moments and the electronic states near the Fermi level are well described within the GGA approximation. Therefore, strong correlation effects are not expected to play a significant role in the electronic structure of the V_2_O-based MOenes studied here. The exchange-correlation (XC) energy was treated within the generalized gradient approximation (GGA) using the Perdew–Burke–Ernzerhof (PBE)^49^ parametrization. van der Waals (vdW) interactions were included through the DFT-D3 method of Grimme.^50^ The electron–ion interactions were described using the Projector-Augmented Wave (PAW) method,^51,52^ with a plane-wave energy cutoff of 500 eV. For geometry optimization, the total energy criterion was set to 1 × 10^–4^ eV, and all the force components are less than 0.01 eV/Å. For phonon dispersion calculations, the energy difference was set to be less than 6.5 × 10^–7^ eV. The Monkhorst–Pack scheme^53^ was used to sample the Brillouin zone, with an 11 × 11 × 1 k-point mesh for structural optimization and a denser 33 × 33 × 1 grid for density of states (DOS) calculations. To avoid interactions between adjacent layers, a vacuum spacing of at least 15 Å was considered along the *z*-direction. Atomic structures were visualized using the VESTA software.^54^

## Results and Discussion

In this section, we present the computed structural and electronic properties of the V_2_O-based MOenes. The dynamic stability was evaluated through phonon calculations, and as a proof of concept, we also calculated the adsorption energy of lithium atoms on the MOene surfaces.

### Structural Properties

Previous studies have reported that the oxidized 1T and 2H–V_2_O are dynamically stable and exhibit metallic behavior, showing promising characteristics for high-performance HER electrocatalysts.^31^ However, neither the pristine systems nor other functional groups have been extensively studied. In this work, we consider both the 1T and 2H polymorphs, the two stable structural configurations commonly reported in MXenes and MOenes,^31,43,55^ for pristine and functionalized V_2_O systems. The surfaces of both structures were functionalized with the chemical species T = {Br, Cl, F, N, OH}.

Figure 1 depicts the optimized atomic structures of the 1T and 2H phases. The calculated lattice parameters for the 1T phase are a = 2.63 Å, the V–O bond length is 2.07 Å, the monolayer height is 2.81 Å, and the bond angles are 78.93° (Figure 1a). For the 2H phase, the optimized lattice constant is a = 2.65 Å, the V–O bond length is 2.10 Å, the monolayer height (h) is 2.83 Å, and the bond angle is 78.82° (Figure 1b). These results are consistent with previous reports on MXenes and MOenes, where the 2H phase generally exhibits slightly larger lattice parameters than the 1T phase.^31,43,56^

**Figure 1:**
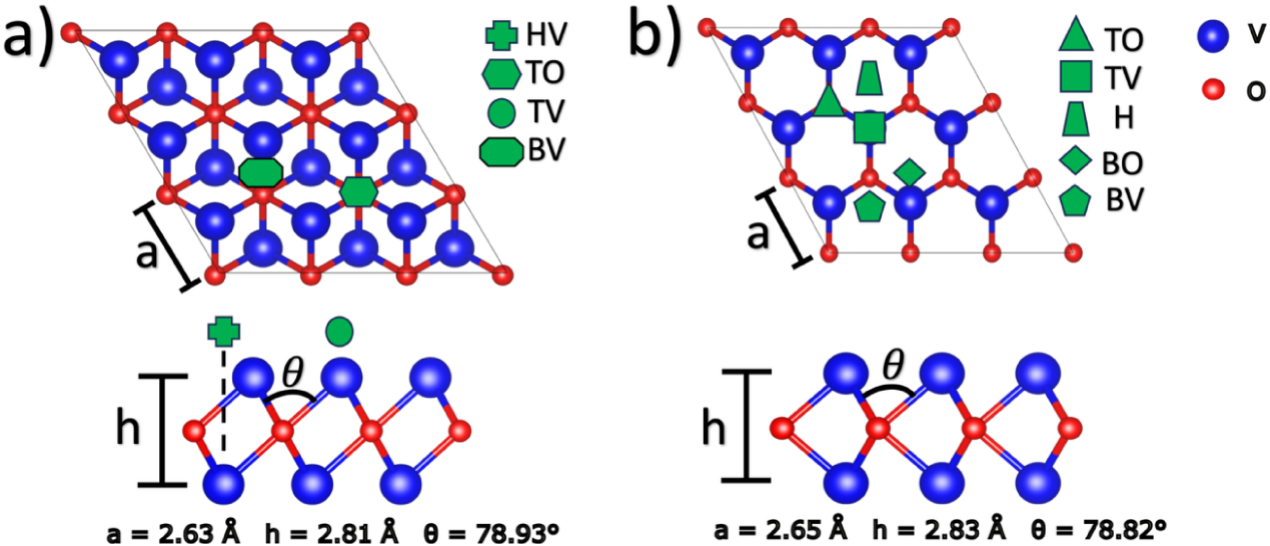
Top (upper panel) and side view (lower panel) of (a) 1T–V_2_O and (b) 2H–V_2_O. The bond lengths for V–O and V–T, and the thickness of the monolayers, are provided. Furthermore, the blue and red spheres denote the Vanadium and Oxygen atoms. The four high symmetry sites for the 1T phase are HV, TO, TV, and BV, and for the 2H phase are TO, TV, H, BO, and BV.

Different high-symmetry adsorption sites were considered for the functionalization of both polymorphs. For the 1T phase, the possible adsorption sites are illustrated in Figure 1a. At the HV site, the functional group is located above the farthest vanadium atom; at the TO site, it is positioned above an oxygen atom; and at the TV site, it is located on top of the nearest vanadium atom. A fourth position, the bridge between two vanadium atoms (BV site), was found to be unstable since the adsorbate consistently relaxes toward the HV site. Therefore, this configuration was excluded from further analysis. For the 2H phase, the possible adsorption sites are depicted in Figure 1b. The TO and TV sites correspond to adsorption above oxygen and vanadium atoms, respectively. The H site represents a hollow site. The BO and BV sites correspond to bridge sites between two oxygen or two vanadium atoms, respectively. However, both bridges were found to be unstable after relaxation, as the adsorbates migrated to the H site.

Figure 2 shows the relative adsorption energies of all considered functional groups at the different high-symmetry adsorption sites for both 1T and 2H phases. This comparison allows us to systematically determine the most energetically favorable configuration in each polymorph. Based on our total-energy calculations, the HV site is identified as the most favorable adsorption site for all functionalized species in the 1T phase (Figure 2a), whereas in the 2H phase, the H site is the most stable configuration (Figure 2b). Consequently, the subsequent Bader charge, structural, and electronic analysis focus exclusively on these most stable configurations, as they represent the most likely structures to be obtained experimentally. Table 1 summarizes the optimized structural parameters (lattice constants, bond lengths, bond angles, and monolayer heights) for the most stable adsorption sites identified in Figure 2, for both pristine and functionalized systems. After full structural relaxation, we compared the lattice parameters, bond lengths, and layer thicknesses of the pristine and functionalized systems. The results show that the adsorption of F and OH groups produces the smallest deviations (less than 5%) from the pristine lattice parameters in both 1T and 2H phases. This minimal distortion indicates that these terminations induce only a weak perturbation to the pristine lattice, thereby enhancing the structural stability of the functionalized MOenes. These findings are consistent with previous reports on Ti_2_O^43^ and V_2_O^31^ MOenes functionalized with O atoms, where structures exhibiting smaller deviations from their pristine geometries were found to be energetically and dynamically more favorable. In contrast, the adsorption of Br, Cl, and N groups leads to greater lattice distortions, which are expected to destabilize the structure by increasing internal strain and inducing a major lattice deformation.

**Figure 2:**
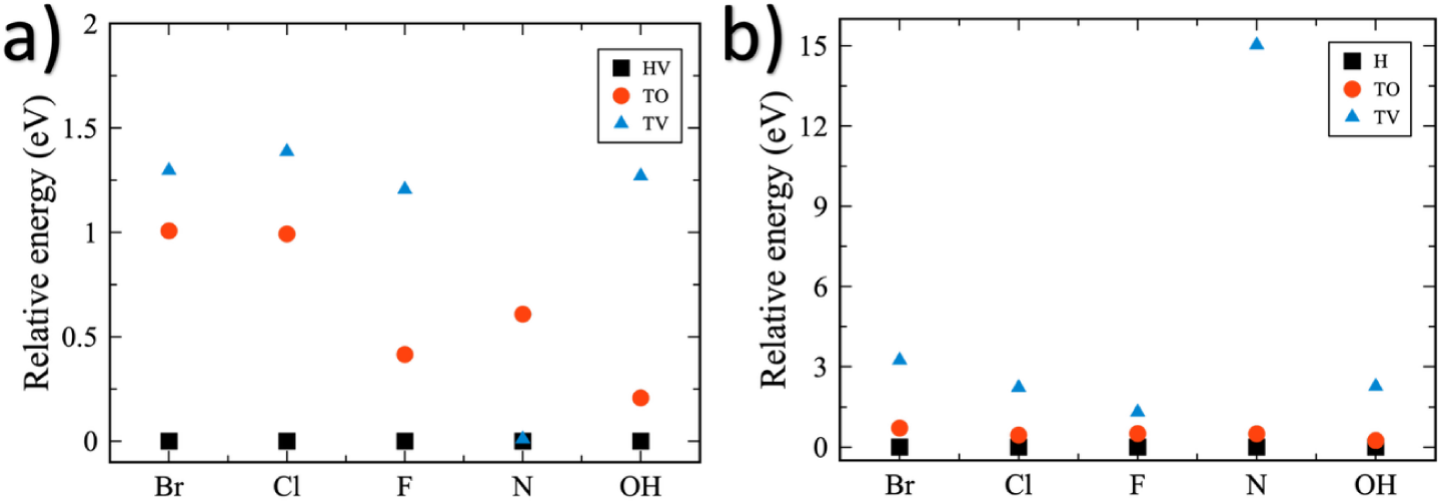
Relative adsorption energies for the functionalization at different high symmetry sites of the (a) 1T and (b) 2H phases of the V_2_O MOene. The most stable site is set to zero as a reference.

**Table 1:** Lattice Parameters, Bond Lengths, Height, and Bond Angles of Pristine and Most Stable Functionalized Sites for the 1T- and 2H–V_2_O-based MOenes^a^

System	a (Å)	V–O bond (Å)	h from V–V (Å)	Bond angle (°)	T–V bond (Å)	h from T–T (Å)
Pristine 1T-V_2_O	2.63	2.07	2.81	78.93	–	–
1T-V_2_OBr_2_	3.33	2.10	1.67	105	2.64	5.29
1T-V_2_OCl_2_	3.25	2.06	1.73	103	2.49	5.01
1T-V_2_OF_2_	2.65	2.11	2.91	77	2.05	5.65
1T-V_2_ON_2_	2.92	2.07	2.93	83	1.80	4.49
1T-V_2_O(OH)_2_	2.71	2.12	2.89	79	2.02	7.43
Pristine 2H–V_2_O	2.65	2.10	2.83	78.82	–	–
2H–V_2_OBr_2_	3.49	2.23	1.93	102	2.60	5.22
2H–V_2_OCl_2_	3.03	2.15	2.51	89	2.37	5.72
2H–V_2_OF_2_	2.67	2.09	2.83	79	2.05	5.56
2H–V_2_ON_2_	2.89	2.22	2.94	81	1.85	4.53
2H–V_2_O(OH)_2_	2.72	2.11	2.82	80	2.02	7.38

a The T represents the functional group T = {Br, Cl, F, N, and OH}.

To further verify the interaction between the functional groups and the MOene surfaces, we performed a charge transfer analysis using the Bader charge method. Table 2 summarizes charge transfer in the pristine structures, serving as a reference for comparison with the functionalized systems.

**Table 2:** Bader Charge Transfer for Each Element in the Pristine and Functionalized MOenes

	V	O	Functional group
1T-pristine	0.60 e	–1.20 e	
1T-F	1.29 e	–1.27 e	–0.66 e
1T–OH	1.40 e	–1.33 e	–1.24 e (O) and 0.49 e (H)
2H-pristine	0.62 e	–1.24 e	
2H–Br	1.19 e	–1.20 e	–0.55 e
2H–F	1.31 e	–1.28 e	–0.67 e
2H–OH	1.40 e	–1.30 e	–1.24 e (O) and 0.59 e (H)

These results confirm the charge redistribution induced by surface functionalization. In particular, the V atoms exhibit greater charge donation than in the pristine systems, indicating a stronger interaction between the MOene surface and the functional groups.

### Dynamical Stability

To assess the dynamical stability of the most stable configurations, we computed their corresponding phonon spectra as shown in Figure 3. The dynamical stability criterion was based on the absence of imaginary vibrational frequencies across the Brillouin zone. As observed in Figure 3a, the pristine 1T phase exhibits very small negative frequencies near the Γ point. However, these values are negligible in magnitude and can be attributed to numerical errors arising from finite grid sampling and convergence thresholds, as reported in other two-dimensional systems.^57–59^ Therefore, the 1T-V_2_O phase can be considered dynamically stable. In contrast, the 2H pristine phase (Figure 3e) is dynamically stable, as all phonon modes exhibit positive vibrational frequencies throughout the entire range of q-points. These results are consistent with previous reports that identify the 2H configuration as the most stable phase both energetically and dynamically.^31,43,56^ Moreover, the stability observed in both pristine 1T and 2H monolayers suggests that similar V_2_O-based MOene structures could remain stable in thicker configurations, such as penta- or hepta-layers, as has been demonstrated in other MXene-like materials.^60^ For the functionalized systems, the phonon spectra reveal that the F- and OH-terminated structures are dynamically stable for both the 1T (Figure 3b and c) and 2H (Figure 3f and h) phases, consistent with the structural analysis presented above. As an illustrative example of an unstable configuration, Figure 3d shows the phonon spectrum of the 1T-V_2_OBr_2_ system, where the presence of several imaginary frequencies across the Brillouin zone indicates dynamical instability. Similar behavior was observed for the other unstable configurations; however, their spectra are not shown since they exhibit similar features. The correlation between phonon stability and reduced lattice distortion further confirms that minimal geometric perturbation enhances vibrational stability in functionalized MOenes. Interestingly, the Br-functionalization 2H system also appears stable, despite inducing significant distortions in the pristine lattice. The small negative frequencies observed in the phonon spectra (Figure 3h) are associated with numerical errors or finite-size effects, as previously reported for other 2D systems.^57–59^ Overall, these results confirm the dynamical stability of the most favorable configurations, suggesting that the experimental synthesis of these V-based MOenes should be feasible.

**Figure 3:**
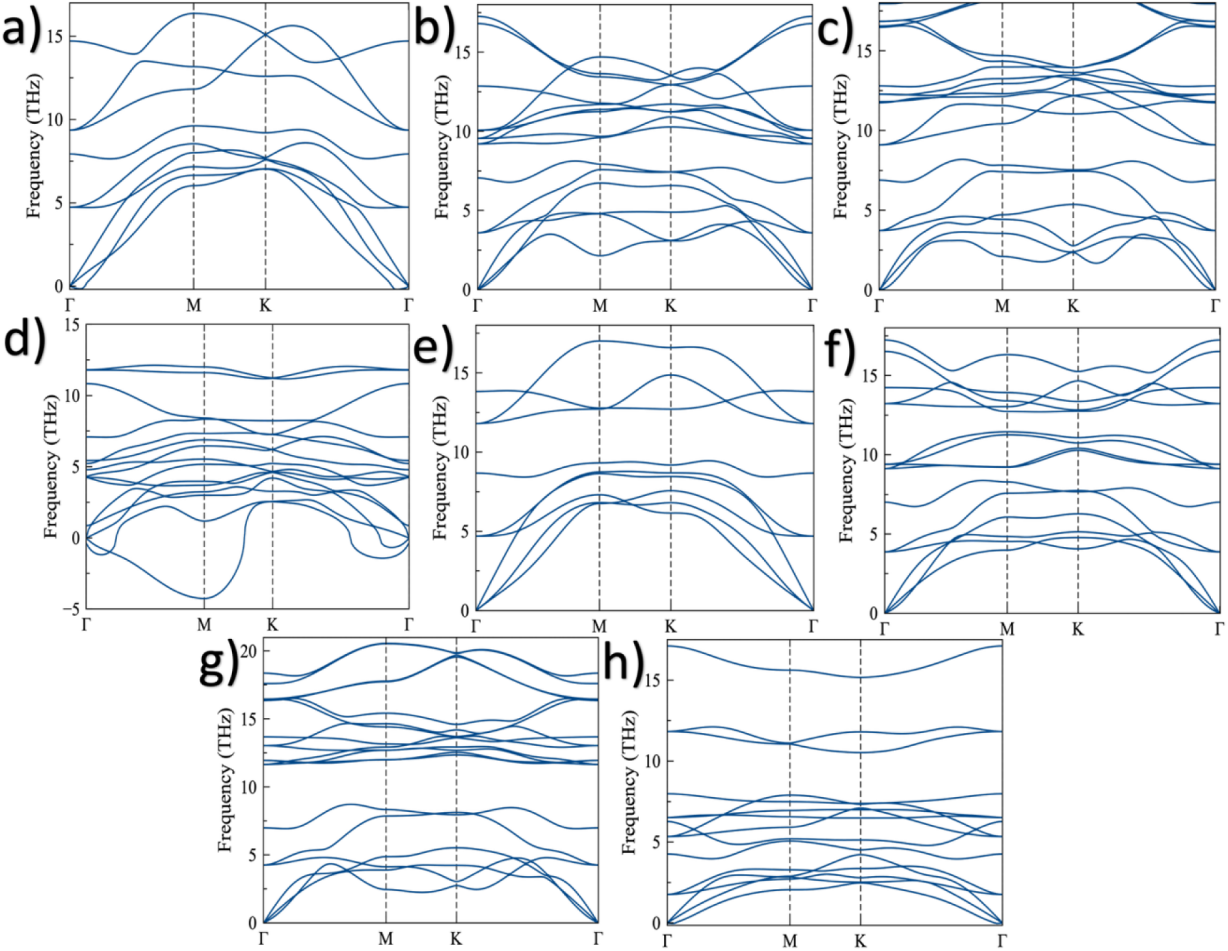
Phonon spectra of V_2_O-based MOenes: (a) pristine 1T phase, (b) 1T–V_2_OF_2_, (c) 1T–V_2_O­(OH)_2_, (d) 1T–V_2_OBr_2_, (e) pristine 2H phase, (f) 2H–V_2_OF_2_, (g) 2H–V_2_O­(OH)_2_, and (h) 2H–V_2_OBr_2_ systems.

### Thermodynamic Stability

To evaluate the thermodynamic stability of the systems, we computed the cohesive and formation energies for each dynamically stable structure. The cohesive energy was calculated as^60–64^

1 $$E_{coh}=\frac{E_{MOene}-\underset{i}{\sum }n_{i}E_{i\left(isolated\right)}}{\underset{i}{\sum }n_{i}}$$

where *E*_*coh*_ is the cohesive energy, *E*_*MOene*_ corresponds to the total energy of the pristine or functionalized system under consideration, *n*_*i*_ is the number of atoms of the *i*-th species (V, O, Br, F, H), and *E*_*i(isolated)*_ is the energy of an isolated atom of the *i*-th species.

The formation energies were calculated as^60,64^

2 $$E_{form}=\frac{E_{MOene}-\underset{i}{\sum }n_{i}\mu_{i}}{\underset{i}{\sum }n_{i}}$$

where *E*_*form*_ is the formation energy, *E*_*MOene*_ and *n*_*i*_ has the same meaning as in eq 1, and μ_*i*_ represents the chemical potential of the *i*-th species. Table 3 summarizes the calculated cohesive and formation energies for the pristine and functionalized MOenes. According to eq 1 and 2, negative values indicate that the structures are thermodynamically stable. The results show that the 2H pristine structure is slightly more thermodynamically stable than the 1T phase. Furthermore, all functionalized systems exhibit negative formation energies, indicating thermodynamic stability, with the 2H-functionalized configurations being the most stable.

**Table 3:** Cohesive and Formation Energies for the Pristine and Functionalized MOenes

	1T		2H	
	E_cohesive_ (eV/atom)	E_formation_ (eV/atom)	E_cohesive_ (eV/atom)	E_formation_ (eV/atom)
Pristine	–6.133	–0.112	–6.195	–0.123
V_2_OBr_2_	-	-	–4.502	–0.117
V_2_OF_2_	–5.481	–0.362	–5.535	–0.366
V_2_O(OH)_2_	–3.876	–0.402	–3.913	–0.405

### Electronic Properties

To study the electronic properties of the 1T and 2H phases of the V_2_O MOene before and after functionalization with thermodynamically and dynamically stable groups, we have computed the electronic band structures and density of states. The calculations reveal that, according to the band structures, both pristine systems behave as conductors (Figure 4a and d). Additionally, the functional F and OH groups do not induce a phase transition to a semiconductor in either phase (Figure 4b, c,e, and f); however, the Br group indeed induces a phase transition to an indirect gap semiconductor, with a bandgap value of approximately 0.64 eV (Figure 4g), a similar behavior is observed in other functionalized MXenes,^62,65^ where different surface terminations significantly modify the electronic structure. More advanced approaches beyond standard DFT, such as hybrid functionals (HSE) or GW corrections, are expected to increase the band gap magnitude to better match experimental results, while generally preserving the qualitative features of the band structure, including the material’s metallic or semiconducting nature. Therefore, while the exact band gap value may change, the predicted metal-to-semiconductor transition induced by Br functionalization is expected to remain valid.

**Figure 4:**
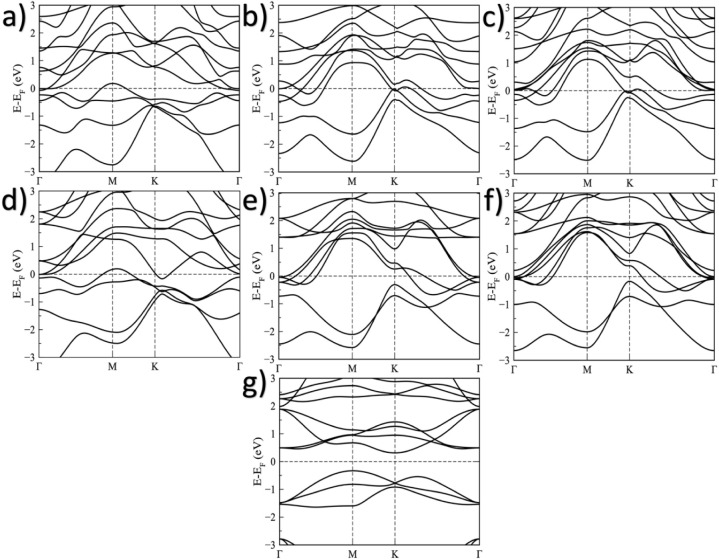
Electronic band structure of the (a) pristine 1T–V_2_O, (b) 1T–V_2_OF_2_, (c) 1T–V_2_O­(OH)_2_, (d) pristine 2H–V_2_O, (e) 2H–V_2_OF_2_, (f) 2H–V_2_O­(OH)_2_, and (g) 2H–V_2_OBr_2_ MOenes.

To further understand the electronic behavior of the most stable configurations, we calculated the projected density of states (PDOS) for the 1T, 2H pristine systems, and for the V_2_OBr_2_–2H system, as shown in Figure 5. We focused only on these three systems because the 1T and 2H functionalized monolayers keep their metallic behavior, except for the Br-functionalized 2H system. These results provide insight into the orbital contributions to the electronic states and help clarify the changes in the band structure, particularly for the Br-functionalized MOene.

**Figure 5:**
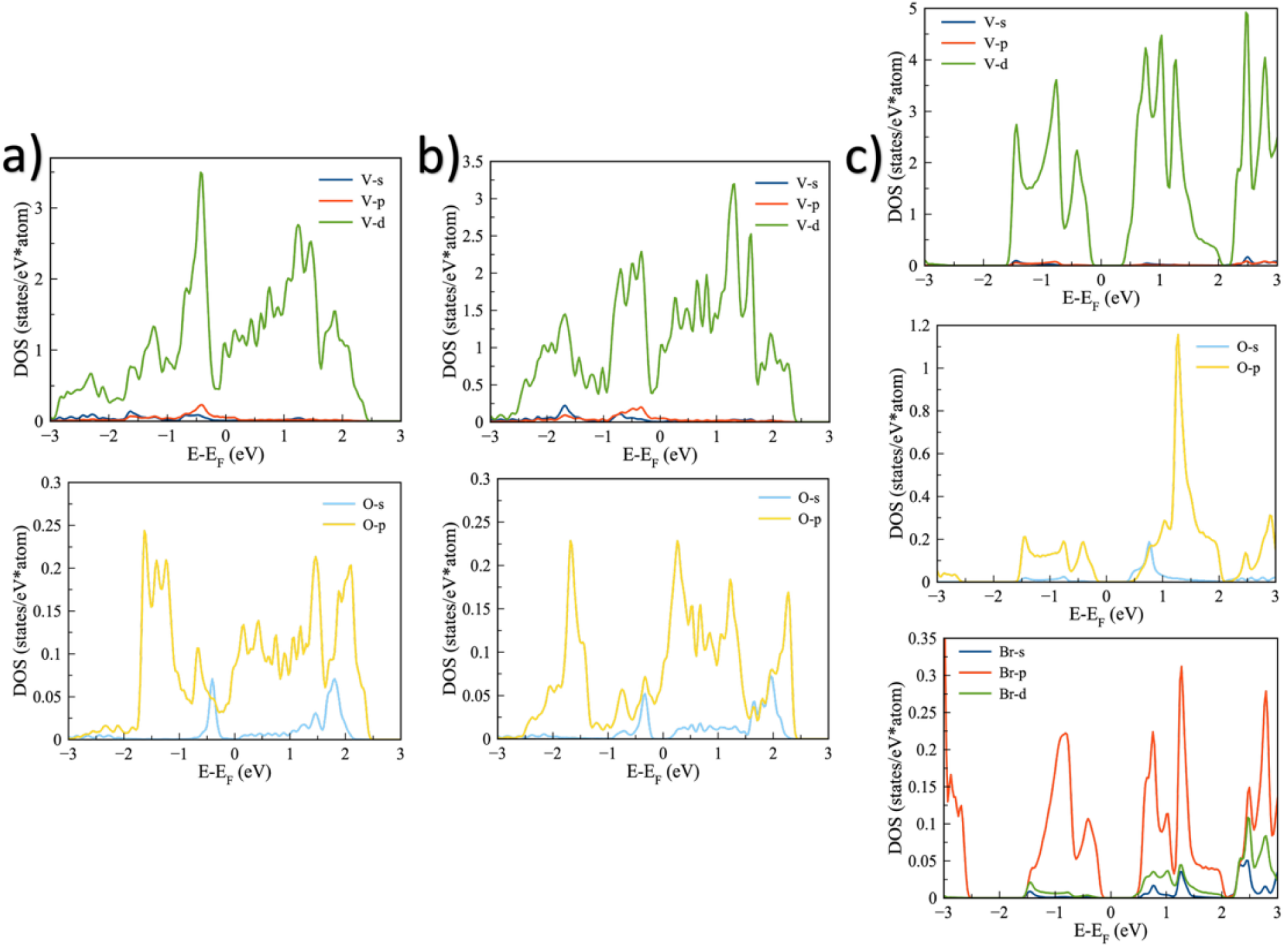
Projected density of states of V_2_O-based MOenes: (a) 1T structure, (b) 2H structure, and (c) V_2_OBr_2_–2H system.

The PDOS of the 1T and 2H phases confirm their metallic character, with the V 3d orbitals dominating the states near the Fermi level. This indicates that vanadium plays the key role in the conduction mechanism for these systems. In contrast, the Br-functionalized systems exhibit a transition from metallic to semiconducting behavior. Although the contribution of Br p-orbitals near the Fermi level is relatively small compared to that of vanadium, their presence modifies the local electronic environment around the V atoms. This leads to a partial redistribution and localization of the V 3d states, therefore opening a small band gap. This transition can be attributed to the stronger electronegativity and larger atomic radius of Br compared to F and OH, which induce local lattice distortions and alter the hybridization between V 3d and O 2p orbitals. The resulting perturbation in the electronic coupling reduces the density of metallic states at the Fermi level, driving the system toward a semiconducting configuration.^66^ These results are consistent with the calculated electronic band structures.

### Li Adsorption

Finally, as a proof of concept, we investigated the adsorption of Li atoms on the most stable surface terminations of the 2H–V_2_O-based MOenes, since this phase has been reported to be the most stable configuration in the TiO_2_ MOenes,^43^ other MXenes,^67^ and in our results. The adsorption energy of Li was calculated as^68–73^

3 $$E_{ads}=E_{V_{2}OT_{2}+Li}-E_{V_{2}OT_{2}}-E_{Li}$$

where *E*_*ads*_ is the adsorption energy of Li atoms on the functionalized V_2_OT_2_ MOene, $E_{V_{2}OT_{2}+Li}$ is the total energy of the optimized Li-MOene system, $E_{V_{2}OT_{2}}$ is the total energy of the isolated functionalized MOene, and *E*_Li_ is the energy of an isolated Li atom.

In the present work, the adsorption energy was calculated using an isolated Li atom as the reference, which is a commonly adopted approach in first-principles studies of ion adsorption on two-dimensional materials.^61–63^ This definition allows us to directly evaluate the interaction strength between the Li atom and the MOene surface.

Using Li metal as the reference corresponds to a different thermodynamic definition, which is typically employed when evaluating formation energies or intercalation potentials in battery materials. In contrast, referencing the isolated Li atom provides a more direct description of the surface adsorption process, which is the primary objective of this part of the study.

The calculated adsorption energies are summarized in Table 4, and the relaxed structures are shown in Figure 6. The V_2_OBr_2_ system exhibits an adsorption energy of −1.54 eV, with a Li–Br interaction distance of 2.68 Å, which slightly elongates the V–Br bond from 2.59 Å to 2.63 Å. For V_2_OF_2_, the adsorption energy is −1.84 eV, and the Li–F distance is 1.80 Å, increasing the V–F bond length from 2.05 Å to 2.18 Å. In contrast, for the V_2_O­(OH)_2_, the adsorption energy is −1.67 eV with a Li–H interaction distance of 2.46 Å. In this case, no significant bond elongation was observed, indicating that the Li atom interacts with the surface without inducing noticeable structural distortion. These results suggest that Li adsorption on V_2_O-based MOenes is energetically favorable, with adsorption energies comparable to those reported for other 2D materials and MXenes used as Li-ion battery anodes.^61–63,74–76^ The moderate adsorption strengths and minimal structural distortion suggest good reversibility and potentially fast ion diffusion,^76^ both desirable features for energy storage applications, particularly in Li-ion batteries.

**Table 4:** Adsorption Energies and Interaction Distances of the 2H–V_2_O Functionalized System and Lithium Atoms

2H–V_2_OS_2_ system	Adsorption energy (eV)	S–Li interaction distance (Å)
V_2_OBr_2_	–1.55	2.68
V_2_OF_2_	–1.84	1.80
V_2_O(OH)_2_	–1.67	2.46

**Figure 6:**
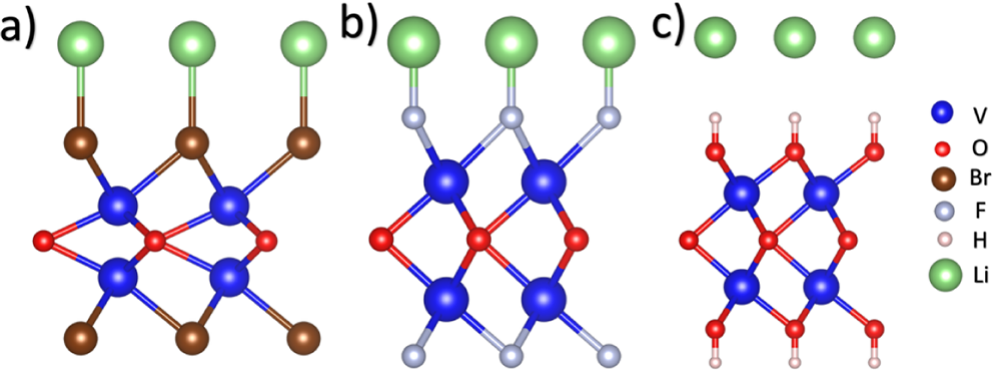
Relaxed structures of (a) 2H–V_2_OBr_2_, (b) 2H–V_2_OF_2_, and (c) 2H–V_2_O­(OH)_2_ interacting with Li atoms.

To further analyze the interaction between Li atoms and the functionalized MOene surfaces, we compute the Bader charge transfer. Table 5 summarizes the charge transfer in the Li adsorption structures for comparison with the results in Table 2. For the V_2_OBr_2_ and V_2_OF_2_ structures, results indicates charge redistribution between the Li atoms and the MOene surface. In contrast, for the V_2_O­(OH)_2_ system, a very small charge transfer is observed between the Li atoms and the surface. Despite the small charge transfer observed in this case, the calculated adsorption energy (−1.67 eV) indicates a moderately strong interaction between Li and the MOene surface. This behavior suggests that the adsorption is not dominated by complete electron transfer but rather by charge polarization and weak orbital hybridization between Li and the surface atoms. Such interactions are favorable for reversible Li-ion storage.

**Table 5:** Bader Charge Transfer for the Li Adsorption Systems

	V	O	Functional group	Li
2H–Br	1.14 e	–1.26 e	–0.71 e	0.20 e
2H–F	1.15 e	–1.25 e	–0.80 e	0.28 e
2H–OH	1.45 e	–1.31 e	–1.24 e (O) and 0.45 e (H)	0.014 e

## Conclusions

First-principles calculations were performed to explore the structural, electronic, thermodynamic, and dynamical properties of pristine and functionalized V_2_O MOenes in both 1T and 2H configurations. Our results demonstrate that the pristine 1T and 2H V_2_O MOenes are dynamically and thermodynamically stable, filling an existing gap in the understanding of this emerging 2D oxide family. The F- and OH-functionalized structures also exhibit both dynamical and thermodynamic stability in the 1T and 2H phases, whereas Br-terminated structures are stable only in the 2H configuration. Interestingly, the Br functionalization induces a transition from metallic to indirect semiconducting behavior in the 2H phase, with an estimated band gap of approximately 0.64 eV. The electronic structure is dominated by V 3d orbitals near the Fermi level, with Br functionalization modulating the density of states and electronic character for the transition. Lithium adsorption studies further confirm the 2H phase as the most favorable surface, with adsorption energies ranging from −1.8 to −1.5 eV and minimal structural deformation. Overall, these findings establish V_2_O-based MOenes as intrinsically stable and electronically versatile materials, holding strong promise for applications in Li-ion batteries and catalysis.
